# Recommendations for Virtual Administration of the PSP Rating Scale

**DOI:** 10.1002/mds.29142

**Published:** 2022-07-10

**Authors:** Anne‐Marie Wills, Lawrence I. Golbe, Anthony E. Lang, Tao Xie, Marian L. Dale, Alberto Espay, Maria Carmela Tartaglia, Susan H. Fox, Sotirios Andreas Parashos, Nikolaus R. McFarland, Ruth B. Schneider, Federico Rodriguez‐Porcel, Steven A. Gunzler, Alexander Pantelyat

**Affiliations:** ^1^ Department of Neurology Massachusetts General Hospital, Harvard Medical School Boston Massachusetts USA; ^2^ Rutgers Robert Wood Johnson Medical School New Brunswick New Jersey USA; ^3^ Edmond J. Safra Program in Parkinson's Disease, Rossy Progressive Supranuclear Palsy Centre Movement Disorders Clinic, Krembil Brain Institute, Toronto Western Hospital, University Health Network and Division of Neurology, University of Toronto Toronto Ontario Canada; ^4^ Movement Disorder Program, Department of Neurology University of Chicago Medicine Chicago Illinois USA; ^5^ Department of Neurology Oregon Health and Science University Portland Oregon USA; ^6^ James J. and Joan A. Gardner Family Center for Parkinson's Disease and Movement Disorders, Department of Neurology University of Cincinnati Cincinnati Ohio USA; ^7^ Tanz Centre for Research in Neurodegenerative Diseases, Rossy Progressive Supranuclear Palsy Centre University of Toronto, Krembil Brain Institute, University Health Network Toronto Ontario Canada; ^8^ Struthers Parkinson's Center, HealthPartners Golden Valley Minnesota USA; ^9^ University of Florida College of Medicine Center for Movement Disorders and Neurorestoration Department of Neurology Gainesville Florida USA; ^10^ University of Rochester, Neurology Rochester New York USA; ^11^ Department of Neurology Medical University of South Carolina Charleston South Carolina USA; ^12^ Parkinson's and Movement Disorders Center, Neurological Institute, University Hospitals Cleveland Medical Center and Case Western Reserve University Cleveland Ohio USA; ^13^ Johns Hopkins University School of Medicine Department of Neurology Baltimore Maryland USA

Our article validating a subset of the Progressive Supranuclear Palsy Rating Scale (PSPRS) for video administration^1^ has prompted questions about how best to administer the individual test items given the constraints of that medium and of the disease. In response, we wish to provide several recommendations below as guidance (Table 1).

**TABLE 1:** Guidelines for virtual administration of the mPSPRS‐21^1^

PSPRS questions. Bolded if removed from the mPSPRS‐21	Guidance for virtual administration
General	We recommend following the guidance provided by the International Parkinson and Movement Disorder Society on optimizing the physical video setup.^2^ If sound quality is poor, consider switching to a telephone for audio to assess voice and speech (while muting your microphone and speaker on the virtual platform). We recommend that the caregiver angles their camera to show the patient's entire body during the motor assessments, which may require moving the camera away from the patient or onto the floor.
11. Grasping/imitative/ utilizing behavior	Ask the caregiver: “Does the patient grab onto the arms of chairs, peoples' arms, clothing, or tablecloths, inappropriately? Do they have difficulty letting go?” 1 = Slight or equivocal; 2 = Definitely present but no effect on daily activities; 3 = mild interference with daily activities; 4 = severe interference, interferes with feeding and dressing.
13. Dysphagia	Have the patient say “Ha, ha, ha” before and after doing the test to see if guttural noises sound wet or gurgling. If wet or gurgling at baseline, do not provide water and score item as 3.
**IV. Ocular motor**	Oculomotor testing can be very difficult to assess by video and therefore has been eliminated from the virtual mPSPRS‐21 version of the scale.
**14. Voluntary upward command movement**	
**15. Voluntary downward command movement**	
**16. Voluntary left and right command movement**	
**18. Limb rigidity**	Excluded from virtual mPSPRS‐21.
**19. Limb dystonia**	As the entire body may not be visible on camera with adequate resolution, this item is also excluded from the mPSPRS‐21.
21. Toe tapping	If the device is a desktop computer, ask the caregiver to move the patient's chair so that the legs are visible and position the patient in profile. If the device is a laptop or tablet, ask the caregiver to aim it at the patient's feet, placing it on the floor if necessary.
**24. Neck rigidity or dystonia**	Excluded from virtual mPSPRS‐21.
25. Arising from chair	Use a non‐wheeled chair without a deeply cushioned seat. The caregiver or a wall should be behind the chair to prevent tipping backwards as the patient sits down. If the patient cannot resist using the arms of the chair, provide an armless chair. Ask the caregiver to guard the patient to prevent falls.
26. Gait	This should be performed under the supervision of a caregiver who is comfortable preventing falls. A gait belt may be helpful. Use a hallway or open area if available, put the camera on the floor, and then ask the caregiver to guard the patient without touching them as they walk to prevent falls. The caregiver, in order to remain free to prevent falls, should not hold the camera during gait testing. If the caregiver feels that the patient is at high risk of falling, or must use a walker to prevent falls, score the item as 3 (must use assistance all or almost all the time).
**27. Postural stability**	Excluded from virtual mPSPRS‐21.
28. Sitting down	Use a non‐wheeled chair. The caregiver or a wall should be behind the chair to prevent tipping backwards as the patient sits down. If the patient cannot resist using the arms of the chair, provide an armless chair.

In summary, we believe that the majority of PSPRS items can be safely and accurately performed via video visits with minimal adjustments. This is of particular importance for patients who cannot travel to the clinical site because of distance, advanced motor disability, lack of transportation, or infectious disease constraints.

## Funding Sources

The Geraldine A. Dolce Fund for Progressive Supranuclear Palsy (PSP) at Massachusetts General Hospital.

## Author Roles

(1) Research project: A. Conception, B. Organization, C. Execution; (2) Statistical analysis: A. Design, B. Execution, C. Review and critique; (3) Manuscript: A. Writing of the First Draft, B. Review and Critique.

A.M.W.: 1A, 1B, 1C, 3A

L.I.G.: 1A, 1C, 3B

A.E.L.: 1A, 1C, 3B

T.X.: 1C, 3B

M.D.: 1C, 3B

A.E.: 1C, 3B

M.C.T.: 1C, 3B

S.H.F.: 1C, 3B

S.A.P.: 1C, 3B

N.R.M.: 1C, 3B

R.B.S.: 1C, 3B

F.R.P.: 1C, 3B.

S.A.G.: 1C, 3B

A.P.: 1A, 1B, 1C, 3B

## Financial Disclosures/Conflict of Interest

The authors have no relevant conflicts of interest.

## Data Availability

Data sharing is not applicable to this article as no new data were created or analyzed in this study.
